# Diversity in medical education: the Indian Paradox

**DOI:** 10.3402/meo.v19.26395

**Published:** 2014-12-10

**Authors:** Sanjib K. Ghosh, Sudipa Biswas

**Affiliations:** Department of Anatomy, ESI-PGIMSR & ESIC Medical College, Kolkata, India

As a country of many ethnic groups and regional variations, the essence of diversity in Indian culture is appropriately reflected in the composition of medical students in any undergraduate training program. Ironically, however, these same diversities that exist in the country's medical education system are also a major barrier in implementing a uniform curriculum. The areas of concern include (1) educational funding and (2) social ‘habitus’ (e.g., personality, interactional skills, perceptions of learning, language).

In India, the costs involved in medical education are borne largely by the state and central governments (1). However, not surprisingly, the ‘economic health’ of states varies considerably, which limits the annual budgets for their respective medical colleges (2). This diversity in available capital, in turn, markedly affects the educational infrastructure and, subsequently, the selection of students seeking to study medicine. Although some preference is given to ‘in-state’ applicants, ample provisions are available for students to attend medical schools in any Indian state they wish. Thus, meritorious students gravitate to best-equipped premier (i.e., well-funded) institutions that provide them with the clinical training needed to practice modern medicine. The divergence of educational capital expended on medical training, it seems, ultimately leads to differential quality of health care for Indian people residing in different parts of the country.

The concept of ‘habitus’ can be understood as a set of socially learned dispositions, skills, and ways of feeling acquired through the activities and experiences of everyday life (3). In India, students in a medical school (irrespective of their merit) come from diverse geographical and social backgrounds – exposing them to different cultures, rituals, customs, and principles (4). Hence, considerable diversity exists among students within medical schools in terms of ‘habitus’ and, accordingly, their interactions with the external environment follow a set pattern guided by their social outlook. Cultural diversity, although commonly seen as a boon for education, can sometimes generate more heat than light (5), which, due to caste-based prejudice and discrimination, is exactly the case in Indian medical schools. This leads to limitations in classroom interaction, exchange of ideas, and dissemination of knowledge – disrupting students’ overall learning and acquisition of crucial clinical skills.

Cultural upbringing, lifestyle, values, and dispositions contribute to students’ social identities, which have significant impact on how they interact – a pivotal aspect of the learning process (6, 7). In India, the diverse social identities among students in a medical school – a result of socio-economic and cultural influences – have a direct bearing on the implementation of the medical education curricula as well as delivery of health care.

In most Indian medical schools, the first and second years of instruction emphasize teacher-centered, classroom-oriented learning (i.e., lectures) – the results of which limit opportunities for interactive learning. Students who rely on memorization and prefer reading text books and class notes typically enjoy this part of the medical curriculum (8). However, from the M3 year onward, the focus shifts from classroom to bedside teaching – where the emphasis shifts to interactive sessions, small group discussions, and cooperation (rather than competition). Here, learners who prefer in-depth understanding of a subject and the application of knowledge tend to excel (9). Such differences in learning ultimately translate into knowledge disparities, potentially adversely affecting the quality of Indian medical education.

Language, too, has a fundamental role in building patient–doctor relationship, the keystone of medical education (10). Linguistically, India has an extraordinary complex array of languages – with differing dialects from region-to-region within a state (11). This is another potential communication barrier for medical students, the most serious effects of which are observed at the clinical level when speaking with patients – a situation sometimes requiring interpreters. At best, such barriers promote an unnecessarily challenging environment for medical students to undertake their clinical training.

In India, provisions exist in every medical school to accommodate different sections of society (12), including students from less affluent areas of the country. The cost of an Indian medical education is still within the reach of even those in the lower socio-economic groups and almost all social strata are represented in any given medical class. Moreover, institutions of higher education have percolated to most rural areas of the country, enabling an intersection of among medical students from urban, suburban, and rural areas of India. Nevertheless, unequal medical school funding and traditional, age-old social practices are persistent cultural forces in the progress and success of Indian medical education. At first blush, the diverse population of medical professionals in India could (and should) be an asset for delivering health care within this vast and culturally diverse population. Ironically, however, a judicious balancing of this diversity with respect to medical education may be required to enhance the quality and societal impact of doctors being trained.

*Sanjib K. Ghosh* Department of Anatomy ESI-PGIMSR & ESIC Medical College Kolkata India Email: drsanjib79@gmail.com *Sudipa Biswas* Department of Anatomy ESI-PGIMSR & ESIC Medical College Kolkata India
